# Obituary

**Published:** 1859-01

**Authors:** 


[COMMUNICATED].

**OBITUARY.**

Died, at the residence of his father, in Henry County, Georgia, on the 9th of September, 1558, after a short illness, Marion M. Dodson, (son of William and Anna Dodson,) aged 23 years, 8 months, and 8 days.

The subject of this notice, was a young man of sterling moral worth, and beloved by all who knew him.

He had attended a course of Lectures in the Medical College of Georgia, and was still prosecuting his Medical studies, bidding fair to become a useful Physician.

His generous nature and gentle manners, won him devoted friends whilst at College, and will ever render his memory in death, sacred to them, as well as all others with whom he associated in life.

Marion M. Dodson, was indeed a virtuous and intelligent young man, and an ornament to society. B.

